# The Effect of Cuelure on Attracting and Feeding Behavior in *Zeugodacus tau* (Walker) (Diptera: Tephritidae)

**DOI:** 10.3390/insects14110836

**Published:** 2023-10-25

**Authors:** Xuxiang Liu, Qinyuan Zhang, Weijie Xu, Yongbang Yang, Qingwen Fan, Qinge Ji

**Affiliations:** 1 Institute of Biological Control, Fujian Agriculture and Forestry University, Fuzhou 350002, China; liuxx1003@163.com (X.L.); congcong9758@163.com (Q.Z.); 15160287616@163.com (W.X.); yyb1234560904@163.com (Y.Y.); 18336086200@163.com (Q.F.); 2 The Joint FAO/IAEA Division Cooperation Center for Fruit Fly Control in China, Fuzhou 350002, China; 3 Key Lab of Biopesticide and Chemical Biology, Ministry of Education, Fuzhou 350002, China; 4 State Key Laboratory of Ecological Pest Control for Fujian and Taiwan Crops, Fuzhou 350002, China

**Keywords:** *Zeugodacus tau* (Walker), cuelure, male lure, feeding, biological control

## Abstract

**Simple Summary:**

The wide distribution of fruit flies and, the diversity of their host species, along with the seriousness of the damage and the huge economic losses they cause, mean that controlling fruit flies is time-critical and economically important. As a highly effective and environmentally friendly method of pest control, lures play an important role in the management of fruit flies. Cuelure (4-(4-acetoxyphenyl)-2-butanone, CL), a male fly lure, was first shown to be attractive to melon flies in 1960. CL is an effective bioactive compound that is commonly used for the investigation, monitoring and trapping of *Zeugodacus tau* Male flies are attracted to the lure, which they then feed on. Here, we tested the effects of fly age and time of day on the response of male *Z. tau* to CL, and the effect of CL feeding on the quality of virgin *Z. tau* males. The results of this study will provide a basic theoretical guide to investigating the tropism mechanism of CL-attracted *Z. tau*.

**Abstract:**

As a vital pest control strategy, trapping plays an important role in the system of monitoring, catching and killing fruit flies. Cuelure (4-(4-acetoxyphenyl)-2-butanone, CL) is a male lure that attracts *Zeugodacus tau* and also stimulates feeding in this species. In this study, the attraction of *Z. tau* to CL and its subsequent feeding behavior were investigated. Under the significant influence of age and time of day, the attraction of CL to *Z. tau* was found to be optimal when flies were 14 days old, and the number of flies trapped increased with trapping duration. It was determined that consumption can improve the mating success and female adult fertility of *Z. tau*. After the observation period, the mating success rate of flies that ingested CL was significantly higher than that of the control group and was maintained at a higher level. It was found that parental consumption of CL could accelerate the development of eggs and larvae, resulting in increased pupation and emergence rates. The results of this study will further clarify the dynamic relationship between pest and lure, and provide a research basis for navigating the integrated management of *Z. tau* in the field.

## 1. Introduction

Except for extreme areas such as deserts and polar regions, fruit flies are widely distributed in almost all biogeographic regions [1,2,3]. Fruit flies mainly feed on plant secretions, nectars, fruits, juices, honeydews, guano and microorganisms [4,5,6]. There are a number of economically important genera (e.g., *Anastrepha*, *Bactrocera*, *Ceratitis*, *Dacus*, *Neoceratitis*, *Rhagoletis*, *Toxotrypana*, *Zeugodacus*) and multiple invasive fruit fly pests (e.g., *B. dorsalis*, *B. tryoni*, *C. capitata*, *Z. cucurbitae*, *Z. tau*) in many countries and regions [7,8,9,10,11,12,13,14]. They not only directly harm fruits and vegetables, but also affect trade circulation to a certain extent. Therefore, quarantine treatment is required for fruit and vegetable commodities before import and export, leading to increased costs for post-harvest protection and entry quarantine. Moreover, the continuous expansion of global trade is facilitating the spread and dissemination of fruit flies worldwide [15,16].

*Zeugodacus tau* (Walker) (Diptera: Tephritidae: *Zeugodacus*) was first discovered in Fujian, China, and reported by Walker in 1849 [17,18,19]. *Z. tau* is widely distributed in Southeast Asia and the South Pacific, including China, India, Korea, Vietnam, Myanmar, Thailand, Laos, Bhutan, Japan, Cambodia, the Philippines, Malaysia, Sri Lanka, Indonesia and other countries [17,20,21]. In China, it is mainly found in Fujian, Jiangxi, Taiwan, Guangdong, Hainan, Zhejiang, Yunnan, Guizhou, Sichuan, Guangxi, Hunan, Hubei, Henan, Shanxi, Gansu and other provinces. The host species in South China are diverse, and the fly is highly abundant and causes serious damage, especially to the fruitlets of solanaceous and cucurbitaceous vegetables [22,23,24,25]. The adults pierce their oviposition tubes into the skin of the fruit and penetrate deeply into the flesh to lay eggs, commonly gathering them in egg piles, and the larvae hatch and develop by feeding on the flesh of the fruit; therefore, the insect mainly damages fruit through larval burrowing. The pericarp of harmed fruit turns brown and black, and in severe cases, the fruit is often eaten away, almost completely rotting and losing its economic value. Less damaged fruit will result in poor growth, causing deformities, and affecting its quality and economic value [6,13,17,18,22,26,27]. Due to its wide range of hosts, vigorous fecundity, great adaptability and ability to cause severe harm, *Z. tau* has been listed as a key quarantine insect in many countries and regions [28,29,30].

The pest management of agricultural crops has a long history of relying on chemical pesticides that are destructive to the ecological environment, which has greatly accelerated the development of environmentally friendly pest control strategies. However, few alternatives can achieve the same efficacy as pesticides. Of course, this includes not only the shelf life of the products but also a series of important factors, such as production costs [31]. Short and long-distance-based pest behavior manipulation methods, including long-distance visual, auditory and olfactory (volatile chemicals) stimuli, as well as the short-distance stimuli of involatile chemicals, provide a practical approach to pest management. Insect behavior results from the integration of various inputs from the central nervous system, which are derived from stimuli acting on multiple receptors (exteroceptors, enteroceptors, proprioceptors). Therefore, achieving adjustments and changes in insect behavior through changes in stimulus is a feasible strategy. The selection of stimuli for behavioral manipulation depends on a number of attributes, according to which we can infer a reasonable order of receptor sensitivity; that is, exteroceptors are more sensitive than enteroceptors [32]. As a pest control strategy based on an olfactory response (exteroceptor-based), lures play a vital role in the management of fruit fly pests.

Lures can stimulate the behavioral response of fruit flies to a certain extent, and play a central role in the sustainable control of fruit flies [31,33,34,35]. The response of flies to lure stimulation varies with the pest’s age and time of day. Since *Z. tau* is an important fly pest, the urgency and importance of controlling it are increasing. The use of male lures to control fruit flies provides possibilities for the sustainable control of *Z. tau*. Male lures, as one of the most important lures, are derived from plant volatiles or parapheromones, and have a significant attractant effect on male flies. Cuelure (4-(4-acetoxyphenyl)-2-butanone, CL) is a male lure with significant effects on *Z. tau*, but it can be used to attract an extensive number of species, including *B. albistrigata*, *B. breviaculeus*, *B. caudata*, *B. curvipennis*, *B. distincta*, *B. facialis*, *B. frauenfeldi*, *B. kirki*, *B. melanota*, *B. obliqua*, *B. psiidi*, *B. tryoni*, *D. bivittatus*, *D. longicornis*, *D. punctatifrons* and *Z. cucurbitae* [36,37,38,39]. Therefore, it is necessary to determine the influence of insect age and time of day on *Z. tau* attracted to CL.

Virgin males respond to CL in the same way as they respond to pheromones [40,41]. There are some similarities between the periodic variation in the trapping effect and the daily rhythm variation in semiochemical release by male fruit flies [42,43]. This may be closely related to the natural presence of CL, which has been confirmed by an increasing number of studies [44,45,46,47]. Therefore, while lures have basic attractant functions, more studies have begun to combine semiochemicals with lures to explore the evolutionary roots of the differences in the responses of fruit flies to lures, and how lures affect the physiology and behavior of fruit flies, to further investigate the multidisciplinary effects of lures [48,49]. Previous studies have shown that male fruit flies are attracted to male lures and feed on them [50]. The explanation for the powerful attraction to and feeding on semiochemicals in males is that fruit flies feed on these for the synthesis of pheromones and thus gain mating advantages [51,52,53]. Male flies are not only attracted by the scent of lures, but they also feed on them. After being ingested, male lure is metabolized into different substances in the male adult body, which then carry out their corresponding functions [13,54,55,56,57,58,59]. Studies on the relationship between CL and different species of fruit flies have mainly focused on *B. tryoni*, *Z. cucurbitae* and *Z. tau*, which are all important pests that can seriously harm most fruits and vegetables [60,61,62,63,64]. The feeding responses of different types of flies vary due to sexual maturity and other factors. Males that have fed on lures will display different responses. Feeding will affect male attraction to both sexes and mating success rate [65,66,67,68,69], horizontal transfer of pesticides or other toxic active substances between males and females [70] and the sensitivity of males to lures [70,71,72,73].

Feeding on lures by virgin males can not only reduce their susceptibility to lures after sexual maturity but can also make their offspring more sensitive to lures, which may contribute to the higher mating success rate of their offspring, thus being beneficial to the reproduction and survival of the fly population [74,75]. However, until now, no studies refer to the effects of feeding lures on the virgin *Z. tau* male before sexual maturity and mating. Therefore, this study mainly includes two aspects. The effects of fly age and time of day on the response to CL by male *Z. tau* adults were measured, based on the assumption that CL is quite attractive to *Z. tau*. Second, the effects of feeding on the growth, development and reproductive parameters of *Z. tau* were tested. The results of this study will provide basic theoretical guidance for exploring the attraction mechanism of CL in male *Z. tau* adults.

## 2. Materials and Methods

### 2.1. Insects

*Z. tau* were reared under controlled temperature, humidity and light conditions (25 ± 1 °C, 65 ± 5% RH, 12:12 h (L:D)) at the Joint FAO/IAEA Division Cooperation Center for Fruit Fly Control in Fuzhou, China. Adults were fed a mixture of sucrose and yeast powder at a ratio of 3:1. In order to ensure fresh food and clean water, and to avoid mildew adversely affecting insect sources, food and water were well supplied and replaced regularly. Gauze cages (L × W × H = 30.00 cm × 30.00 cm × 30.00 cm) were used, covered on six sides with 100 mesh nylon, with one side with a cylindrical operating cuff of approximately 30.00 cm in length and 10.00 cm in diameter to facilitate the test operations inside [76,77,78].

### 2.2. The Effect of Time of Day and Fly Age on Male Z. tau Adults’ Attraction to CL

Twenty-five male *Z. tau* adults of different ages (10, 12, 14, 16, 18, 20 d) were selected and, respectively, put into a different test cage (L × W × H = 30.00 cm × 30.00 cm × 30.00 cm) used for each age 60 minutes before the experiment began to adapt to the environment.

#### 2.2.1. The Effect of Time of Day and Fly Age on the Attraction of Male *Z. tau* Adults to CL

A trap bottle (d: 6.00 cm, h: 13.00 cm) containing 1.00 mL CL was placed in the bioassay cage, while a trap bottle without CL was installed for a separate control group (CK). The test time consisted of three periods (09:00–12:00, 14:00–17:00, 19:00–22:00) each day, and each period was tested separately for 3 h using different fly sources. After the test period, the number of *Z. tau* individuals in the trap bottle was counted (Z), and the data were used to calculate the attractive rate of *Z. tau* (A%) under different ages and times of day. A% = Z/25. Each treatment was replicated four times.

#### 2.2.2. The Effect of Duration of Exposure and Age on the Number of *Z. tau* Adults Attracted to CL

The test duration was 3, 6, 9 and 12 h (started at 09:00). The control group was a trap bottle without CL. The same conditions and statistical methods were used as above (Section 2.2.1). Data were used to calculate the attractive rate of CL to *Z. tau* under different ages and trapping durations. Each treatment was replicated four times.

#### 2.2.3. Feeding Behavior of *Z. tau* Adults on CL

One male adult *Z. tau* was selected and placed in the bioassay cage. Round filter paper (d: 7.00 cm) coated with 100 μL CL (not in the control group) was placed in a Petri dish (d: 9.00 cm) in the center of the cage. The length of time before feeding, the duration of feeding time and the total number of feeding attempts were recorded. The observation time was at the same time each morning (10:30–11:00) and the total observation duration was 30 min. Each treatment was replicated ten times.

### 2.3. Effects of CL Consumption on Male Z. tau Adults

The pupae of *Z. tau* were separated into 3.00mL finger tubes, and both male and female adults were reared separately after emergence. Representative 14-day-old virgin male *Z. tau* adults were randomly selected from a test cage. Filter paper (d: 7.00 cm) coated with CL was placed in the center of the cage for feeding *Z. tau* at different test periods (A.M. 8:00–8:30, A.N. 12:00–12:30, P.M. 16:00–16:30). In the control group (CK), 14-day-old male and female virgin *Z. tau* that had not been fed on CL were selected. After feeding, 50 *Z. tau* were collected in different cages over three test periods. The number of *Z. tau* deaths within 30 min after treatment was counted to calculate the short-term mortality of male adults. Without a supply of any food or water, the number of dead *Z. tau* was counted every 8 h to calculate the survival of male adults under stress. Each treatment was replicated three times.

An equal number of 20 treated male and female adults of the same age were kept in a cage with a supply of food and water. Mating was observed and the number of pairs mating was counted every 10 min. When the adults successfully mated, the mating pair was carefully removed with a finger tube until the test was completed. We analyzed the trend in the number of mating pairs over time and the mating success rate of the *Z. tau* adults. Testing was conducted from 17:00 to 19:00 over a period of 2 h. Each treatment was replicated five times.

A total of 20 mated pairs of 14-day-old *Z. tau* adults were placed in a cage with a supply of food and water which was replaced every other day. In one part (20 pairs), the number of dead *Z. tau* adults was counted each day, and the dead were collected to calculate the death rate (D %) and lifespan (L) of *Z. tau*. D% = A/20. L = (L1 + L2 + … + L20)/20. Male and female adults were counted separately. In the other part (20 pairs), fresh pumpkin was used to collect the eggs once every other day over a duration of 30 min (16:00–16:30). Eggs were collected five times in total, and the number of eggs collected each time was counted to calculate female fecundity (F). F = number of eggs under stereomicroscope. Each treatment was replicated five times.

Subsamples of eggs were randomly selected and sandwiched between two fresh slices of pumpkin, which were placed in a sterile Petri dish to prevent the eggs from drying out. Larvae were placed on freshly sliced pumpkin in Petri dishes on fine sand to pupate when the larvae matured. The pupae were placed in the adult room. The number of eggs, larvae and pupae was 50 per replicate. The frequency of egg hatching (under a microscope), larval pupation and pupal emergence were observed every 3 h to calculate the length of the developmental period (D) and the rate (R%) of each at different stages. D = (D1 + D2 + … + Dn)/n. R% = (number of egg hatching/larval pupation/pupal emergence)/50. In order to measure pupae weight, 50 pupae were randomly selected and individually weighed using an electronic scale. The number of female and male adults emerging from 50 pupae was counted to calculate the ratio of females to males. Each treatment was replicated five times.

### 2.4. Data Analysis

WPS Office 2022 (Kingsoft Co., Ltd., Beijing, China) was used to collect the raw data, calculate the indicators and produce Figures and graphs. All data were analyzed using an analysis of variance (ANOVA) with SPSS v.23.0 (SPSS Inc., Chicago, IL, USA), and multiple comparisons were performed using the Duncan method. Statistical results were expressed as mean ± SD, with *p* < 0.05 considered statistically significant, and multiple comparison results were marked using the letter-marking method.

## 3. Results

### 3.1. Reaction of Different-Aged Male Z. tau Adults to CL

#### 3.1.1. The Effect of Time of Day and Fly Age on Male *Z. tau* Adults’ Attraction to CL

**Figure 1:**
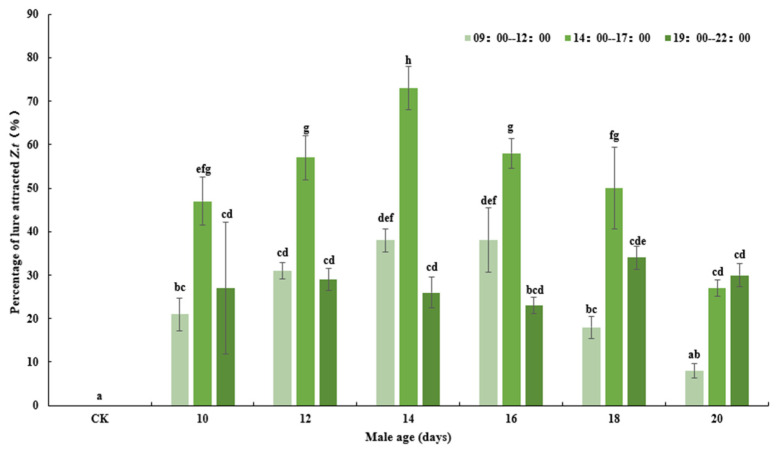
Effect of age and time of day on the percentage of CL–attracted *Z. tau*. Here, and below, bars refer to mean ± SE and different letters above the histograms indicate significant differences (Duncan’s methods, *p* < 0.05). CK means control with no treatment.

According to experimental results and data analysis, the age of the flies (*F*_6, 63_ = 27.663, *p* < 0.001), the time of day (*F*_2, 63_ = 42.706, *p* < 0.001) and the interaction between these two variables (*F*_12, 63_ = 4.230, *p* < 0.001) all significantly affected the attraction of CL to *Z. tau* (Figure 1). A greater number of 14-day-old *Z. tau* individuals were attracted to the CL than individuals of other ages, and the difference was significant between 14-day-old and 10- and 20-day-old *Z. tau* (*p* = 0.030, *p* < 0.001). When the adult males were 10, 12, 14 and 16 days old, the number of trapped *Z. tau* in the afternoon was significantly higher than that in the morning and evening (*p* = 0.004, *p* = 0.036; *p* = 0.004, *p* = 0.002; *p* < 0.001, *p* < 0.001; *p* = 0.036, *p* < 0.001). The number of *Z. tau* attracted to lures in the afternoon was significantly higher than that in the morning and evening, and the difference between them was significant (*p* < 0.001, *p* < 0.001). The attraction effectiveness in the morning and afternoon increased with the age of *Z. tau*, reaching its peak at 14 days old.

#### 3.1.2. The Effect of Duration of Exposure and Age on the Number of *Z. tau* Adults Attracted to CL

**Figure 2:**
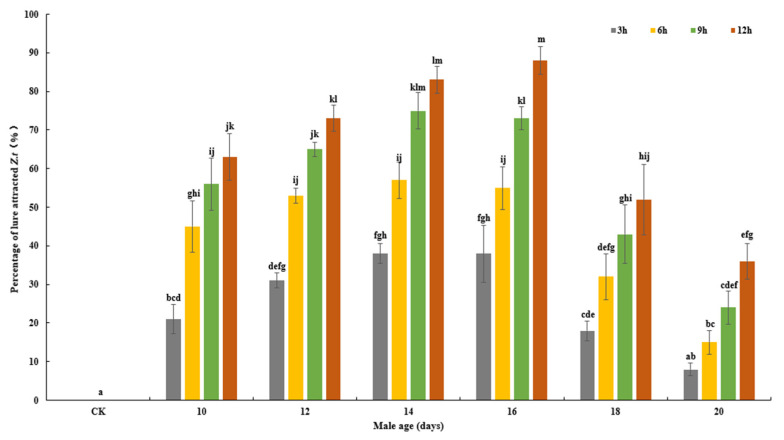
Effect of age and trapping duration on the percentage of CL–attracted *Z. tau*.

The results show that the number of trapped *Z. tau* increased over time. The main effect age (*F*_6, 84_ = 110.553, *p* < 0.001), trapping duration (*F*_3, 84_ = 74.628, *p* < 0.001) and interaction between these variables (*F*_18, 84_ = 2.948, *p* < 0.001) significantly affected the CL attraction effectiveness on *Z. tau* (Figure 2). With the increase in age, the increase in the number of trapped *Z. tau* at 3 to 6 h and at 6 to 9 h was slower, while the number at 9 to 12 h was increased.

#### 3.1.3. Feeding Behavior of Different-Aged Male *Z. tau* Adults on CL

**Figure 3:**
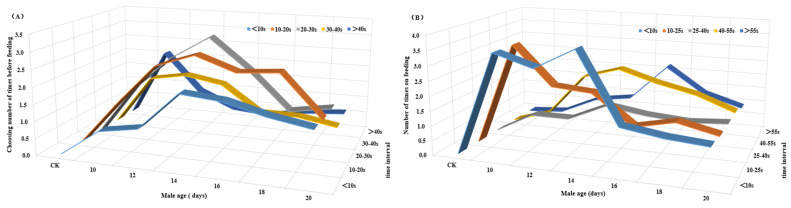
Age affected the number of times before (**A**)/on (**B**) feeding of CL–attracted *Z. tau*.

The main effects of age (*F*_6, 315_ = 22.855, *p* < 0.001), selection time before feeding (*F*_4, 315_ = 19.630, *p* < 0.001) and the interaction between these variables (*F*_24, 315_ = 4.025, *p* < 0.001) significantly affected the selection times under a given feeding time (Figure 3A). The main effects of age (*F*_6, 315_ = 22.450, *p* < 0.001), feeding duration (*F*_4, 315_ = 18.636, *p* < 0.001) and the interaction between these variables (*F*_24, 315_ = 6.126, *p* < 0.001) significantly affected feeding times under a given feeding duration (Figure 3B).

**Figure 4:**
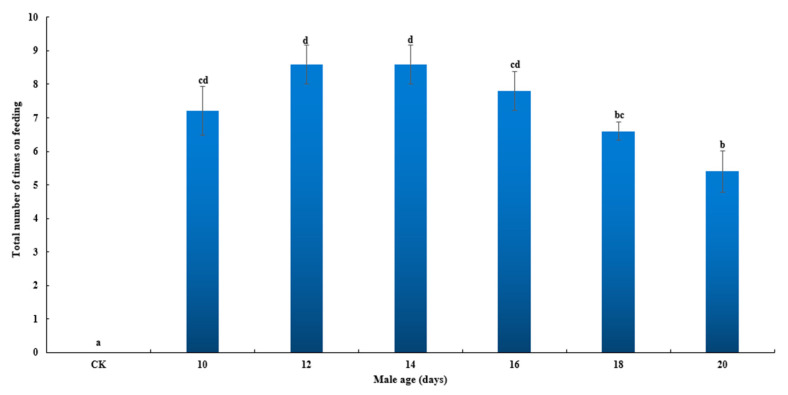
Age affected the total number of feeding times of CL–attracted *Z. tau*.

The results showed that the age of the flies significantly affected the total number of CL feeding times of *Z. tau* (*F*_6, 63_ = 31.846, *p* < 0.001, Figure 4). The total number of feeding times of *Z. tau* at 10, 12, 14, 16, 18 and 20 days old were significantly higher than for the control group (*p* < 0.001, *p* < 0.001, *p* < 0.001, *p* < 0.001, *p* < 0.001, *p* < 0.001). The total number of feeding times of *Z. tau* at 10 and 16 days old was significantly higher than at 20 days old (*p* = 0.020, *p* = 0.002), and the total number of feeding times of *Z. tau* at 12 and 14 days old was significantly higher than at 18 (*p* = 0.010, *p* = 0.010) and 20 days old (*p* < 0.001, *p* < 0.001).

### 3.2. Effects of CL Consumption on Male Z. tau Adults

#### 3.2.1. Short-Term Mortality and Survival under Stress

**Figure 5:**
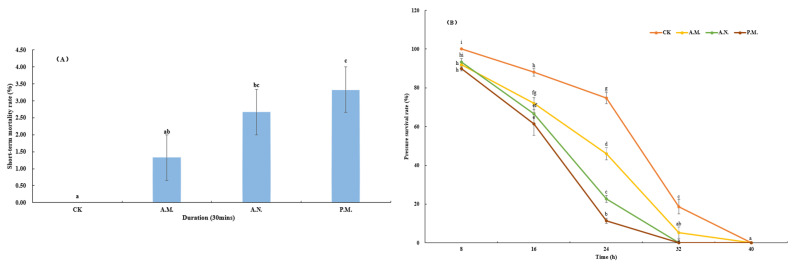
Effect of feeding on short-term mortality rate (**A**) and survival under stress (**B**) of *Z. tau*.

The feeding treatment significantly affected the short-term mortality of *Z. tau* (*F*_3, 8_ = 6.556, *p* = 0.015, Figure 5A), and the number of deaths at noontime and in the afternoon was significantly higher than for the control group (*p* = 0.011, *p* = 0.004). The number of deaths was significantly lower in the morning than in the afternoon (*p* = 0.040). Regarding survival under stress, the main effects of the feeding time slot (*F*_3, 40_ = 83.829, *p* < 0.001), observation duration (*F*_4, 40_ = 1033.100, *p* < 0.001) and interaction between these variables (*F*_12, 40_ = 18.357, *p* < 0.001) significantly influenced the *Z. tau* index (Figure 5B). There were significant differences in survival under stress between the treatment and control groups (*p* < 0.001, *p* < 0.001, *p* < 0.001); there were also significant differences between morning time and noontime or afternoon (*p* = 0.001, *p* < 0.001).

#### 3.2.2. Mating Success Number and Rate

**Figure 6:**
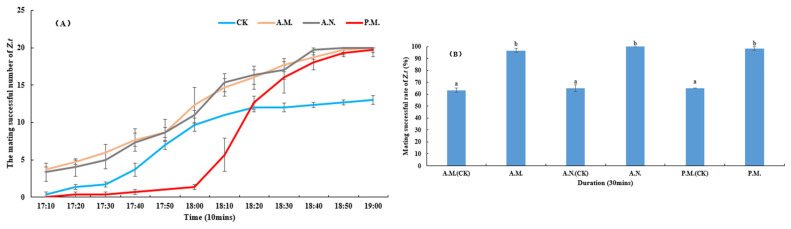
Effect of feeding on the mating success number (**A**) and rate (**B**) of *Z. tau*.

The *Z. tau* mating success number was significantly affected by the feeding time slot (*F*_3, 144_ = 85.992, *p* < 0.001), observation timing (*F*_11, 144_ = 178.306, *p* < 0.001) and interaction between these variables (*F*_33, 144_ = 5.295, *p* < 0.001, Figure 6A). The rate of mating during morning and noontime in the treatment group was significantly different from the control group (*p* < 0.001, *p* < 0.001), and in the afternoon it was significantly different from in the morning and noontime (*p* < 0.001, *p* < 0.001). The feeding treatment significantly affected the breeding success rate of *Z. tau* (*F*_5, 12_ = 124.567, *p* < 0.001, Figure 6B), and the index of the feeding group was significantly higher than that of the control group (*p* < 0.001, *p* < 0.001, *p* < 0.001).

#### 3.2.3. Mortality, Adult Lifespan and Female Fecundity

**Figure 7:**
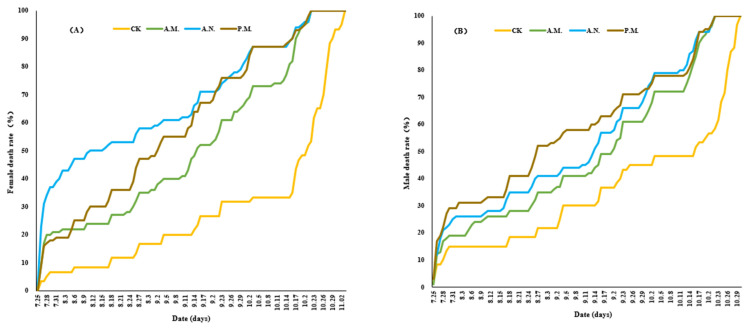
Effect of feeding on the female (**A**) and male (**B**) *Z. tau* death rate with time.

The CL treatment increased the mortality of male and female *Z. tau* adults after mating, and the effect of noontime and afternoon feeding on adult mortality was more significant than that of the morning (Figure 7A,B).

**Figure 8:**
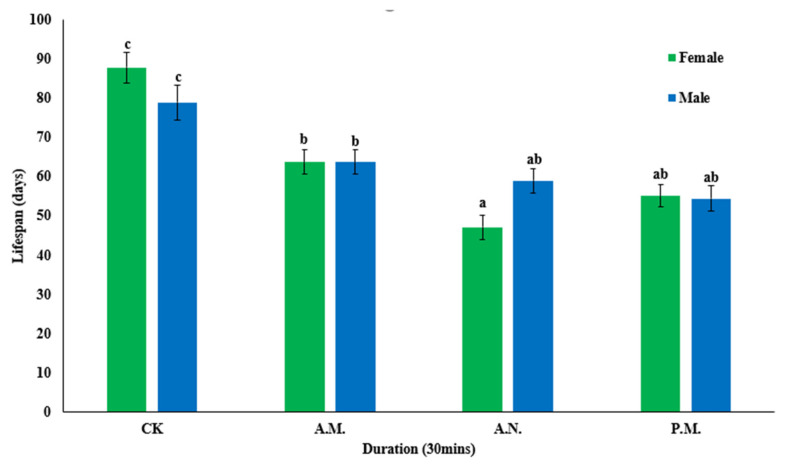
Effect of feeding on the female and male lifespan of *Z. tau*.

The feeding treatment significantly reduced the lifespan of male (*F*_3, 356_ = 7.946, *p* < 0.001) and female (*F*_3, 356_ = 24.361, *p* < 0.001) *Z. tau* adults after mating (Figure 8). The lifespan of female (*p* < 0.001, *p* < 0.001, *p* < 0.001) and male (*p* = 0.003, *p* < 0.001, *p* < 0.001) *Z. tau* adults undergoing feeding treatment was significantly lower than that of the control group, and that of female *Z. tau* adults in the morning was significantly higher than that for noontime and the afternoon (*p* < 0.001, *p* = 0.042).

**Figure 9:**
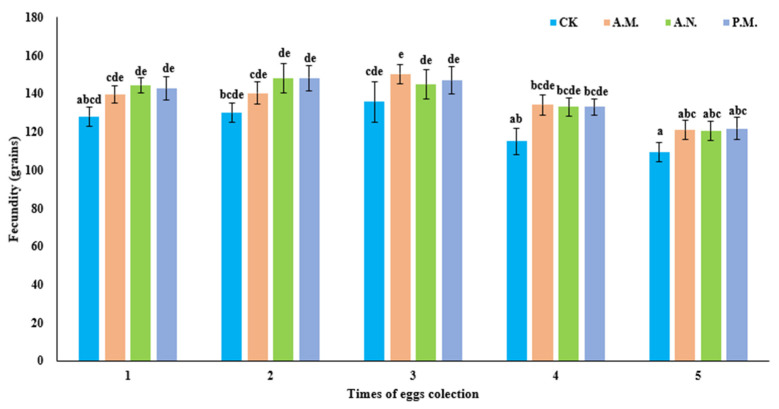
Effect of feeding on the female fecundity of *Z. tau*.

In terms of female fecundity, the feeding time slot (*F*_3, 90_ = 5.293, *p* = 0.002) and time of egg collection (*F*_4, 90_ = 12.233, *p* < 0.001) significantly influenced the fecundity of *Z. tau* (Figure 9). Female fecundity in the treatment group was significantly different from that in the control group (*p* = 0.002, *p* = 0.001, *p* = 0.001). The time of egg collection showed that the fourth was significantly different from the first, second, third and fifth times (*p* = 0.029, *p* = 0.005, *p* = 0.001, *p* = 0.010). The fifth was significantly different from the first, second and third times (*p* < 0.001, *p* < 0.001, *p* < 0.001).

#### 3.2.4. Developmental Duration and Rate of Hatching, Pupation and Emergence, Pupal Weight and Sex Ratio

**Figure 10:**
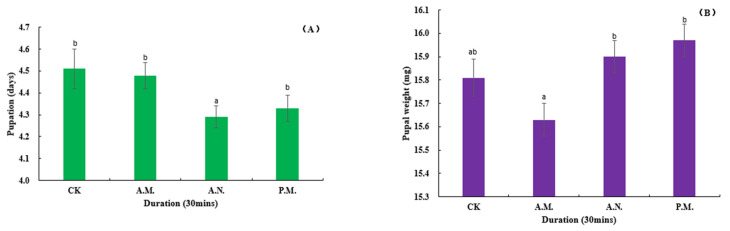
Feeding effect on the pupation development duration (**A**) and pupal weight (**B**) of *Z. tau*.

**Figure 11:**
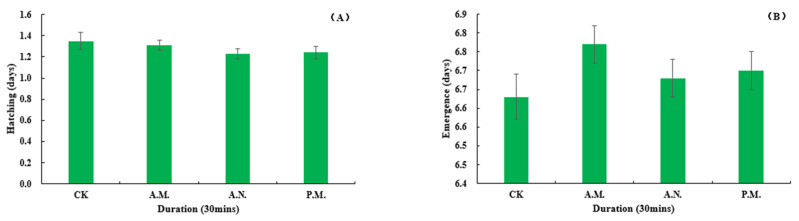
Feeding effect on the development duration of hatching (**A**) and emergence (**B**) of *Z. tau*.

**Figure 12:**
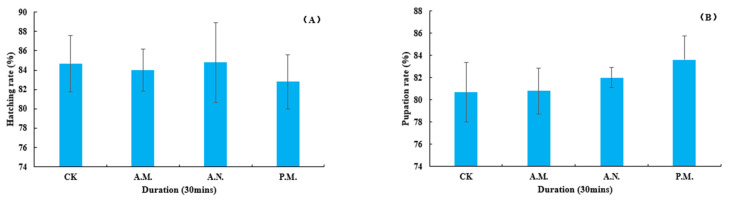
Feeding effect on the rate of hatching (**A**) and pupation (**B**) of *Z. tau*.

**Figure 13:**
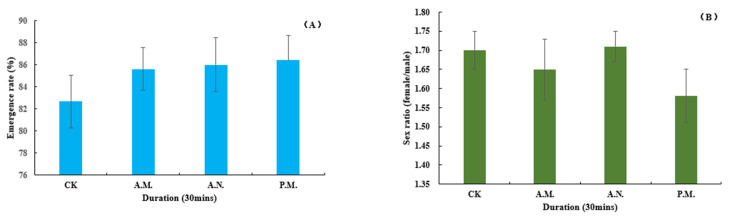
Feeding effect on the emergence rate (**A**) and sex ratio (**B**) of *Z. tau*.

Among the biological indexes of the offspring which parent bred by CL, the developmental duration of pupation (*F*_3, 896_ = 2.957, *p* = 0.032, Figure 10A) and pupal weight (*F*_3, 896_ = 4.865, *p* = 0.002, Figure 10B) were significantly affected by the feeding treatment, while the duration of hatching (*F*_3, 896_ = 0.880, *p* = 0.451, Figure 11A) and emergence (*F*_3, 896_ = 1.069, *p* = 0.361, Figure 11B); the rate of hatching (*F*_3, 14_ = 0.085, *p* = 0.967, Figure 12A), pupation (*F*_3, 14_ = 0.499, *p* = 0.689, Figure 12B) and emergence (*F*_3, 14_ = 0.417, *p* = 0.744, Figure 13A) and the sex ratio (*F*_3, 14_ = 0.872, *p* = 0.479, Figure 13B) were not significantly affected by the treatment.

## 4. Discussion

Lures can be applied in prognosis and prediction, trapping and killing, traditional prevention and control, mating interference and other forms of pest management [79]. The control efficiency of lures on pests is affected by many factors in the actual production process. For example, the best lure performance of *B. tryoni* and *D. cacuminatus* is different for each season. The maximum attraction effect of CL on *B. tryoni* was found to be earlier than for ME (methyl eugenol) on *D. cacuminatus*. The same semiochemical will show different activity to the target insect depending on the season. An earlier study on CL demonstrated that the diurnal attractants of CL to the flies decreased over time in summer, but showed a first rise then descend in spring, with a peak at noon [80]. This is consistent with the results of this test. Fruit flies are not strongly attracted to lures at all times, but have an almost uncontrolled reaction to lures at certain periods, and this change in time-of-day effect may be influenced by the pest age and trapping duration [81,82,83]. Moreover, there is not a simple positive linear correlation between pest age and attraction effect, and the dynamic dialectical relationship between them is affected by many factors [84]. Wong et al. used different strains to determine the effect of age on CL-attracted melon flies, and the results were consistent with the present test, laboratory-reared flies showing a response to CL at age 14 days over 96%. However, we can see that the source of the pest can affect the results of the test. For wild flies, the age that can cause the maximum response is greater than that of laboratory-reared sources, possibly because of the difference in speed of sexual maturation [85]. This difference in the source of the insect was also confirmed in the study by Manoukis et al. [86]. Furthermore, Manoukis et al. used computer vision to test the difference in diurnal rhythmicity of melon flies attraction to CL. Morning and noon are the times when melon flies are strongly attracted to CL. The explanation for this difference is that the daily cycle of attraction of tephritid males to para-pheromone seems to be opposite to the cycle of mating behavior [87]. The results of this study are almost consistent with the results of previous studies, which showed that the effectiveness is greater at noontime for most ages, and different ages and times of day could significantly influence the attraction effect of CL on *Z. tau* [80,85,87]. This phenomenon is closely related to the response rhythm of *Z. tau* to the bioactive compounds in lures. This is similar to the way insects exist in nature with their own rhythms (e.g., circadian rhythm, mating rhythm) [88,89,90,91]. Of course, such periodic transformations in lures may also be an innate response rhythm based on the specifically ontogenetic stages of insects [92].

As a lure that is highly effective on a wide range of insects, CL plays an important role in the green control of flies [37,38]. The monitoring of CL duration can ensure the minimization of prevention and control costs and the maximization of effectiveness. Our experiments derived a generally accepted conclusion that the attractant duration is positively correlated with quantity. However, the experiments were conducted indoors, and their duration seemed short compared with the long period of field trapping trials. The field test cycle is relatively long, so the quality and potency of the lure during use become an important focus [93,94]. Therefore, our tests should explore in depth how to maintain the lure activity over a longer period. Mixing lures on new media (e.g., cotton wicks, coconut husks, caneite blocks, fiberboards, molded paper fibers, and Min-U-Gel) is a highly feasible measure. The loss rate of the bioactive compounds is slowed down and the cost of prevention and management is reduced [95,96,97,98,99]. In the meantime, we should also try to discover lures based on novel media [100,101], (mixed poison/non-toxic) solid lures (e.g., cones, mallets, plugs, strips, tapes, wafers) are emerging, the forms of which are different from traditional liquids. The gradual replacement of liquid by solid lures is a reflection of environmentally friendly prevention and control strategies. This form of CL, including plugs and wafers, displays similar attractant effectiveness to traditional liquid CL. This development innovation can not only greatly improve the control efficiency, but can also reduce the dependence on traditional, highly toxic insecticides, and maximize the diversified needs of disparate agricultural activities for lures [102,103,104,105,106,107,108]. In fact, no matter which medium is used as the basis of the lure, the duration should be the main focus of attention. While considering the prevention and control effect, cost and efficiency are also key elements that managers must focus on when making their choice to add more feasible ways to apply lures in the management of fly pests.

Lures are already widely used in the investigation, monitoring and trapping of fruit flies. It is generally understood that lures attract pests by odor, but more studies have proved that pests can consume lures. At the same time, this feeding phenomenon is similar to the attraction effect of lures on fruit flies, and there may be a certain rhythm to this. Therefore, according to the previous studies and the influence of different ages on the luring effect of CL on male adults, different feeding periods were designed for the experiment. In other words, the efficiency of feeding with lures is closely related to time.

The effect of lures on target pests differs between fly species and stages of sexual maturity. The results of this study indicate that feeding can affect a series of adult *Z. tau* biological indicators. Feeding can increase the short-term death rate of *Z. tau*, which may be related to the over-excited response of male adults after feeding, who then die from consuming a lot of energy. The focus of further research in subsequent experiments should be on whether the excessive mating of male adults is involved [66,109]. The mating incubation period becomes longer with delays in feeding treatment. The morning and noon groups began mating during the initial observation period, and mating occurred in all periods of the observation period, with the distribution relatively scattered. However, in the afternoon group, only a few pairs began to mate in the first half of the observation period, with the mating peak being relatively concentrated. The mating rate between treated male and normal female adults was significantly higher than that in the control group, and the mating success rate in the treatment group was close to 100% at the end of observation. Interestingly, the afternoon period had a lower mating rate at the beginning of the observation period. The mating rate during this time period was closer to the mating peak in the twilight period, and whether excessive feeding doses lead to sluggish movement is an interesting scientific question. Furthermore, feeding on CL can significantly improve the mating success rate of male and female fecundity. Compared with the control group, the mating success rate of *Z. tau* treated at the same time was higher. It is likely that CL-fed males are more attractive than non-fed males when competing for females to mate with; thus, they enjoy a higher mating success rate [65,68]. The increased fecundity of females after mating with CL-fed males may contribute to the reproduction and development of their own populations [73]. It potentially improves the CL foraging and searching ability of male offspring, but there was no obvious improvement in the trapping effect of CL on male adults. This is consistent with previous studies [52,67,72,74,110,111,112].

CL is an effective lure for male flies, which can play a variety of functions in the field of fly pest management. At present, there is a lack of basic studies on the lure control of *Z. tau*. Based on this, the results confirm the effects of age, time of day and trapping duration on CL lures targeting *Z. tau*. At the same time, based on the results of previous studies on the feeding effects of other fly pest species, this study explored the feeding effects of the lure on the lifespan, fertility and other aspects of target pests. In short, CL is an effective lure for pest management, and our study has identified the attracting and feeding relationship between *Z. tau* and CL. The results of this study will further clarify the interaction between *Z. tau* and multifunctional lures, providing guidance for the green control of *Z. tau*.

## 5. Conclusions

According to the experimental results and the above discussion, this study shows that factors such as age, time of day and trapping duration can significantly influence the effectiveness of CL in trapping male adult *Z. tau*. Feeding CL to *Z. tau* has a crucial impact on its fitness. The results of this study provide a theoretical basis for further clarifying the application of feeding behavior in the field of biological pest control. It contributes to optimizing the pest control system, enriching the specific content of the strategy and promoting the development of sustainable plant protection.

## Data Availability

The datasets in this study are available from the corresponding author on reasonable request.
